# Rotating between ponatinib and imatinib temporarily increases the efficacy of imatinib as shown in a chronic myeloid leukaemia model

**DOI:** 10.1038/s41598-022-09048-5

**Published:** 2022-03-25

**Authors:** H. Jonathan G. Lindström, Ran Friedman

**Affiliations:** 1 Department of Chemistry and Biomedical Science, Linnaeus University, Kalmar, 39 182 Sweden; 2 Present Address: Faeth Therapeutics Inc., Oakland, CA USA

**Keywords:** Chronic myeloid leukaemia, Computational biology and bioinformatics, Computational models

## Abstract

Targeted therapies for chronic myeloid leukaemia (CML) are effective, but rarely curative. Patients typically require treatment indefinitely, which gives ample time for drug resistance to evolve. Drug resistance issues are one of the main causes of death owing to CML, thus any means of preventing resistance are of importance. Drug rotations, wherein treatment is switched periodically between different drugs are one such option, and have been theorized to delay the onset of resistance. In vitro testing of drug rotation therapy is a first step towards applying it in animal or human trials. We developed a method for testing drug rotation protocols in CML cell lines based around culturing cells with a moderate amount of inhibitors interspersed with washing procedures and drug swaps. Drug rotations of imatinib and ponatinib were evaluated in a CML specific cell line, KCL-22. The growth of KCL-22 cells was initially reduced by a drug rotation, but the cells eventually adapted to the protocol. Our results show that ponatinib in a drug rotation temporarily sensitizes the cells to imatinib, but the effect is short-lived and is eventually lost after a few treatment cycles. Possible explanations for this observation are discussed.

## Introduction

Chronic myeloid leukaemia (CML) has defined the paradigm of targeted cancer treatment, after the resounding success of imatinib in the early 2000s. These targeted therapies consist of tyrosine kinase inhibitors (TKIs), which eliminate the aberrant kinase activity of Bcr-Abl1. However, the precision with which targeted therapies act means even small changes can lower their effect, rendering them vulnerable to the clonal evolution present in all forms of cancer^1–3^. Unfortunately, even during early trials, reports of some patients growing resistant started to come in^4^. Since then, similar accounts exist for all drugs that are used in CML treatment^5^. Unlike most forms of cancer, CML has a single molecular driver, caused by a chromosomal translocation, that in essence only exists in cancer cells: Bcr-Abl1^6^. For this reason, CML responds so well to targeted treatment, as the sole cause the malignancy can be effectively suppressed targeting only Bcr-Abl1^7^. On the other hand, inhibition of Bcr-Abl1 puts a high evolutionary pressure on the cancer, which is the reason for the development of tens of different resistance mutations. As a consequence of this, a significant fraction of CML patients do relapse with some drug resistance adaptation.

**Figure 1:**
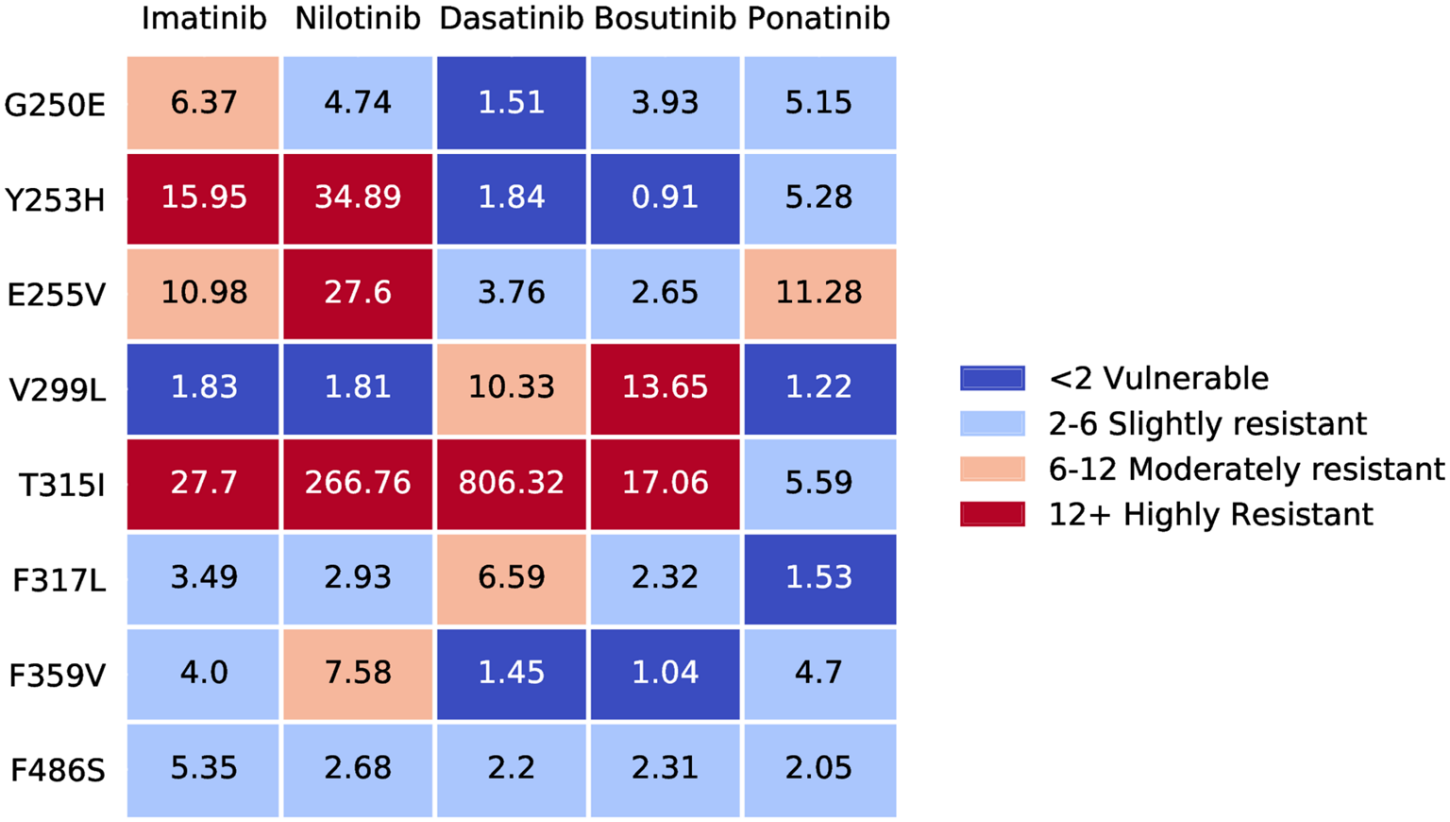
An example of the variability in the effects of a resistance mutation against different drugs. The given numbers are IC50 values relative to the IC50 in cells harbouring a non–mutated Bcr-Abl1, given as multiples of the non–mutated IC50^11^.

Most commonly, resistance occurs through mutations in the kinase domain (KD) of Bcr-Abl1, hindering drug binding and restoring oncogenic signalling^8^. Sensitive diagnosis of mutations is paramount for the success of targeted therapy in CML ^9^. Often times, these mutations specifically provide protection against one or a few drugs (Fig. 1), with the most problematic being the well known T315I gatekeeper mutation. This mutation provides a high degree of resistance towards all drugs except ponatinib^10^.

While mutations and an increased expression of Bcr-Abl1 are the main Bcr-Abl1 dependent resistance mechanisms, other changes in the cancer cells can provide protective effects. Many TKIs are substrates of drug efflux transporters, in particular ABCB1 and ABCG2; an increase in their activity has been associated with resistance^12^. Of note, this does not seem to affect all TKIs, and it has been suggested that TKIs that are not affected by such transporters might be a better first choice drugs ^13^. Another possibility involves replacing and/or supplementing Bcr-Abl1’s role as the singular oncogenic driver. Most commonly this involves abnormal activity of Src. Apparently, some second generation TKIs are also active against Src (dasatinib, bosutinib and ponatinib) which may provide some additional protective effect^14^.

Several approaches have been suggested to limit the scope of drug resistance. The most common approach involves waiting for the earliest sign of relapse, and then attempting to select a drug that should be effective against whatever resistance adaptation may be present. Typically, sequencing of the Bcr-Abl1 KD is performed to identify mutations and select a drug accordingly, which can be beneficial^15^. This methodology is currently applied clinically, albeit not universally^16^. Another approach to reduce the risk of drug resistance is drug combinations^11,17,18^, where two or more drugs are used at the same time. By selecting drugs with complementary mechanisms and non–overlapping resistance adaptations they can be made both robust and efficient. In particular, the novel inhibitor asciminib seems promising in this regard^11,18,19^. However, drug combinations may not be tolerable or may exhibit unpredictable negative interactions. Yet another strategy is to employ drug rotations where the therapy alternates between two or more drugs. The idea is that switching between different drugs periodically should lower the incidence of resistance since a trait that confers resistance towards drug A might not protect against drug B and vice versa. This strategy can be viewed as less beneficial than combination therapy, but do not suffer the same risk of negative interactions^20,21^. We have previously predicted that beneficial drug rotations may exist in CML using currently approved drugs^22^ with computer modelling. For this to work, it is critical that the drugs have a different set of resistance mutations (e.g. Fig. 1). Drug rotations involving ponatinib, which is the only drug effective against the T315I mutation (in the simulation study), were predicted to have the biggest effect.

To estimate the viability of a drug rotation approach as suggested in our earlier study^22^, we exposed KCL-22 to a drug rotation of imatinib and ponatinib (K562 were also tested, but did not survive the therapy despite multiple trials). KCL-22 cells seem to become resistant easily when cultured with a TKI and do not require long term exposure^23^. The cells were cultured in several replicates on a multi well plate in the presence of either imatinib or ponatinib. Drug concentrations were chosen such that the cells would grow at approximately half their normal rate. This made it possible to maintain the cell densities of a normal culturing protocol for each of the cell lines, and to follow their growth through periodic counting. Every six days, the cells were washed to remove the previous inhibitor (or simulate the stress of washing for the control groups), and reseeded with a new inhibitor while maintaining a suitable population density, in a new plate. This was repeated six times, generating a timeline of population size under either a drug rotation protocol, or a monodrug control treatment.

## Results

**Figure 2:**
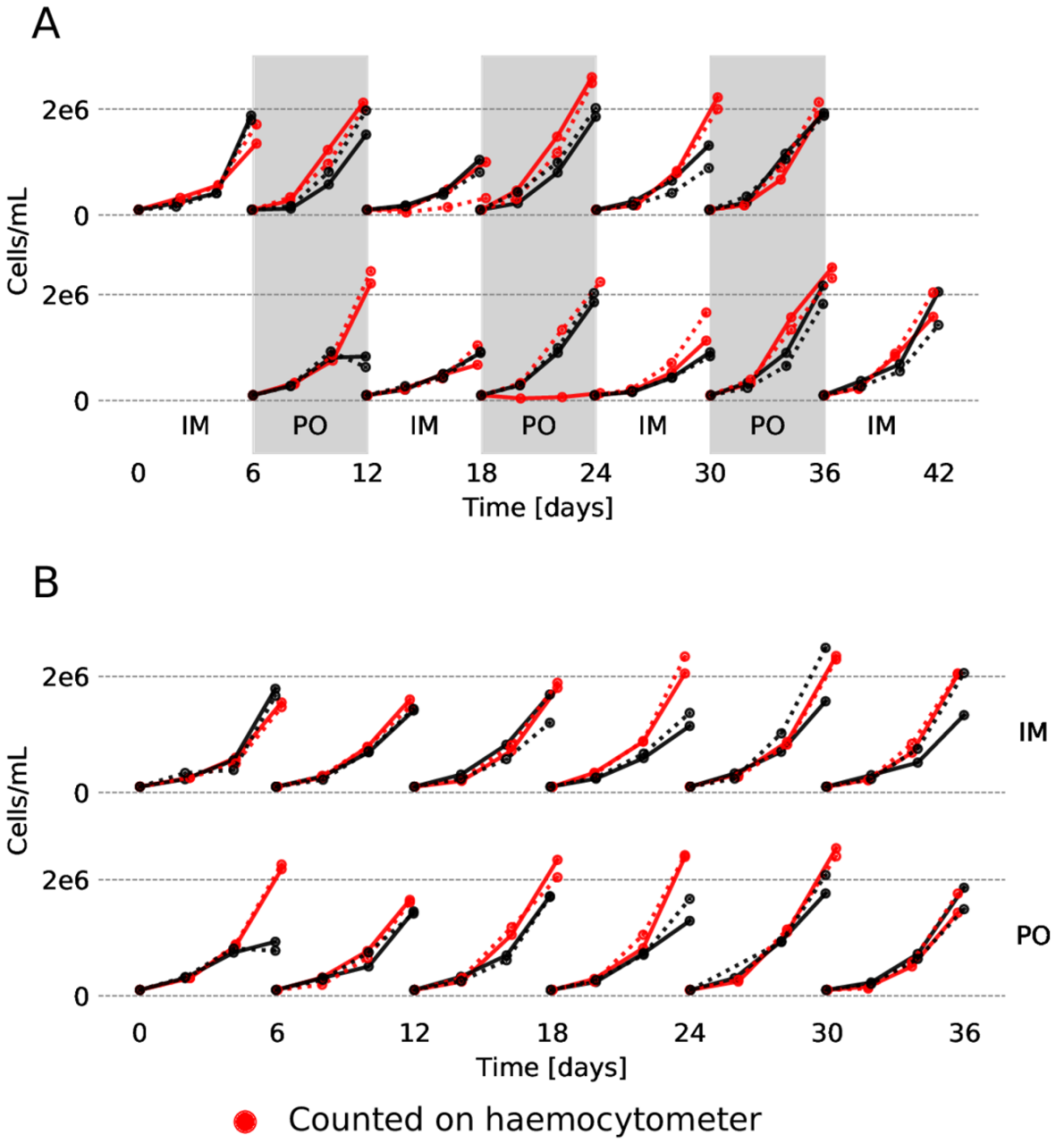
The number of cells (population size) over time for each culture. The timelines are grouped by the treatment. Replicates that were counted manually using a haemocytomer are indicated by red colour. (**A**) Cells exposed to drug rotations, aligned in time such that drug exposure coincides. (**B**) Cells exposed to monodrug therapy (washed every six days but not subject to rotation). The colours and line–styles are consistent between this figure and Fig. S2.

We examined a drug rotation of imatinib and ponatinib since imatinib is the oldest and most frequently used TKI, and ponatinib has the biggest potential in drug rotations out of the currently available options^22^. In addition, imatinib is often better tolerated than many other TKIs whereas ponatinib is associated with cardiovascular toxicity. A rotation protocol in cell lines, developed to this aim^24^, was used. Each drug was used for six days sequentially, after which cells were washed and exposed to either the other drug (rotation groups) or the same drug again (control groups). After the washing procedure, an average of 54% cells remained, which was enough to reseed down to 1 × 10^5^ cells/mL in a majority of cases. A total of six treatment periods were performed (Fig. 2). A target density of 1 × 10^5^ cells/mL was also determined to allow for excellent growth of the cells (Fig. S1) thus making sure that growth during the drug rotation experiments was not limited by an insufficient seeding density.

There are a some events of note in the population size over time graph (Fig. 2). First, the population size during the initial exposure to ponatinib in KCL-22 cells measured using an automated counter diverged from the manual count at the final timepoint. The reason for this is unknown, but as the experiments were not done simultaneously, it may be explained by somewhat changed conditions. Other than that, manual and automated counts produced remarkably consistent results. Second, another unusual observation is in the second exposure of KCL-22 to ponatinib (Fig. 2, red line, second row), where one culture in the manually counted replicate did not grow as expected. This is likely a case of excess cells being lost in the washing procedure, which is why an additional count was introduced for all the automatically counted replicates in both cell lines. Finally (and most interesting), during the imatinib cycle that followed the first ponatinib treatment, the cells appeared to have grown overall worse than expected. This might indicate that the resistance mechanism employed by the cells following ponatinib therapy makes them more sensitive to imatinib, which would not be explained by any known resistance mutation as these would make the cells resistance to both drugs. Imatinib is more effective at suppressing growth once the cells have been exposed to ponatinib, though the effect eventually fades (Fig. S3).

**Figure 3:**
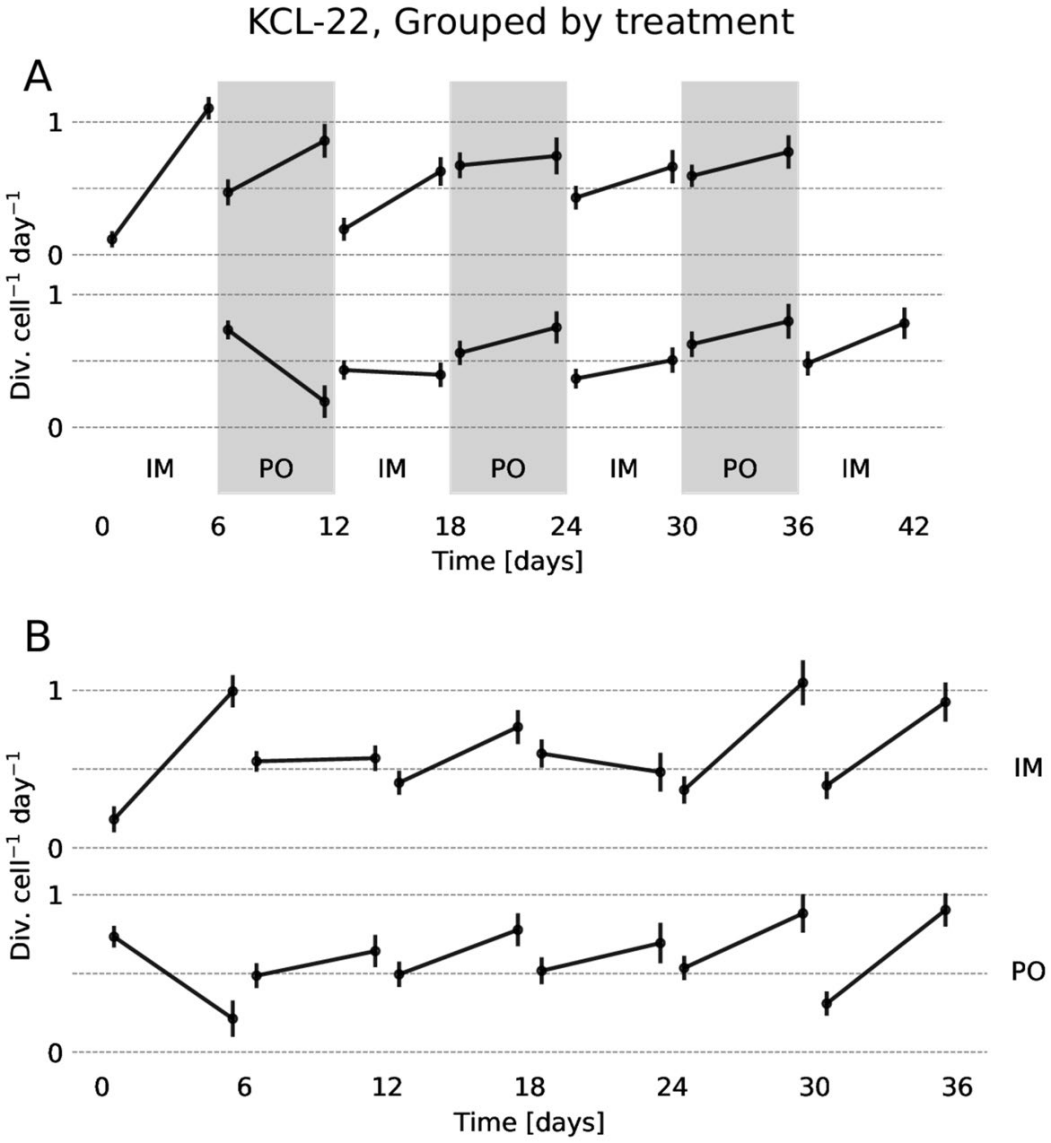
The inferred growth rate over time for KCL-22 cells. The error–bars show the standard deviation, and are inflated somewhat by a degree of multicolinearity between the initial and final growth rates. (**A**) The growth rate over time in cells exposed to drug rotations, aligned in time by the drug exposure. (**B**) The growth rate in cells exposed to monodrug therapy (washed every six days but not subject to drug rotation).

Using the ratrack tool for deriving growth rates^25^ we calculated how the growth rate changed over time during the treatment. A grouping by protocol, and an individual calculation were both performed for each separate well. The results are presented in Fig. 3 and S2–S3. There is a general trend towards growth rates increasing over time, which is an indication of the cells gradually becoming resistant towards the drugs. The most notable exceptions to this progressions are (i) the first treatment the cells are exposed to, and (ii) the first imatinib treatment after a ponatinib period in the drug rotation group. Drug rotations seem to enhance the effect of imatinib, possibly at the cost of the effectiveness of ponatinib, when considering only total growth, as derived by integrating there results (Fig. S3).

**Figure 4:**
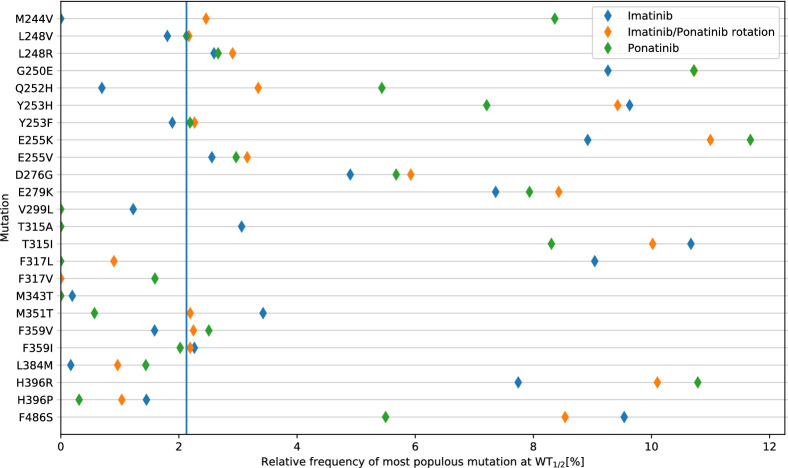
Calculated frequencies of the mutations when half of the population of cancer stem cells is no longer wild-type. Calculations were performed for cells treated with imatinib, ponatinib, or a rotation of the drugs.

To better understand the mutational landscape that is expected for the cells, we simulated how resistance developed using the wollsey tool^22^, studying ponatinib/imatinib rotations explicitly (Fig. 4). As observed, and in agreement with clinical findings, many mutations are forthcoming in the population. The T315I mutation is the most common upon treatment with imatinib. As multiple compound mutations that involve T315I lead also to ponatinib resistance due to increased activity of the enzyme^26^, such mutations presumably lead to the failure of rotation treatment but do not explain the initial higher efficacy of imatinib following ponatinib therapy. Considering that (1) ponatinib generally displays better efficacy for Bcr-Abl1 than for variants that are resistant against imatinib (2) imatinib resistant mutations are numerous and (3) the best chance of eliminating a preexisting mutation is as early as possible, using the best inhibitor (ponatinib) as a temporary first line is advantageous for minimising the risk of drug resistance.

## Discussion

In terms of resistance, ponatinib is the most potent TKI used in CML. Furthermore, it appears to target leukaemia stem cells more effectively than imatinib^27^. However, the drug is not used as first line therapy due to a risk for cardiovascular toxicity. Here, we developed a protocol to study drug rotations in CML cell lines and used it to study a rotation between imatinib and ponatinib. Based on a modelling study where only resistance mutations where considered, we hypothesized that such a rotation would postpone but not terminate the development of pan-resistance mutations, as any single mutation developed by cell exposed to imatinib will be wiped by ponatinib. In agreement with this prediction, drug rotation therapy made KCL-22 CML cells more sensitive to imatinib and less likely to develop imatinib resistance, but this effect was only temporary. Ponatinib has been shown to be an inhibitor, but not a substrate of ABCB1^28,29^. ABCB1 is implicated in imatinib transport, and resistance caused by ABCB1 upregulation following imatinib treatment has been observed^30^. Thus, a reasonable explanation for the effects seen in the drug rotation, is that ponatinib exposure altered ABC-transporter activity in the cells which in turn reduces the ability of these transporters to clear the cells of imatinib. An alternative explanation would be that some ponatinib remains in the cells despite our washing protocol. This explanation is however refuted as in this case cells treated with ponatinib will over time grow slower after washing (as the concentration of the drug increases if it is not thoroughly washed) which is not the case.

In light of the results, pretreatment with a TKI that is less sensitive to drug resistance and later treatment with one that is more sensitive but carries fewer side effects might be beneficial, whereas a rotation protocol might not be useful (with ponatinib and imatinib, in any case, Fig. 2). Of note, a rotation protocol was not successful in cellular models of acute myeloid leukaemia either^24^. Interestingly, a clinical trial (TIPI/NCT04070443) is currently undergoing where ponatinib is used for six months followed by imatinib for at least 30 months. The results are expected to be completed in 2027. Our study gives further hope for the approach that serves a basis to the study, of which we were not aware at the start of our investigation.

## Materials and methods

We used KCL-22 that were a gift from Prof. Leif Stenke. The cell lines were cultured in RPMI-1640 with 10% FBS and 1% Pen–Strep. Cultures were grown at 37 °C, 5% CO_2_ and maintained between 10^4^ cells/mL by dilution every 2 to 3 days. All experiments were initiated with cells of passage < 10, designating the original gifted cells as passage 0. K562 cells were also obtained from the same source, but did not survive therapy and were hence discarded.

### Drug efficacy assay

10^4^ KCL-22 cells per well were seeded in triplicate in 100 μL medium into a 96 well plate prepared in advance with imatinib (7.81nM to 1600nM) or ponatinib (0.0312nM to 64.0nM). The plate was incubated at 37 °C, 5% CO_2_ for 2 days, after which 20 μL MTS (Celltiter 96 AQ_ueous_ One Solution Cell Proliferation Assay, Promega) was added to each well and left to incubate for 3h. Finally, the absorbance at 490nM was measured on a plate reader (Tecan Spark^®^).

### Drug rotations

10^5^ KCL-22 in 1mL cells were seeded in quadruplicate medium in two 24 well plates, in the presence of either imatinib (240nM) or ponatinib (0.37 nM), each in quadruplicate. Each well was counted every other day using an automated cell counter (LUNA-II) and a 1:1 trypan blue (0.4%) stain. After six days, the contents were transferred to Eppendorf tubes and centrifuged at 300*g*. The supernatant was discarded and the pellet resuspended in medium, before being spun down again and resuspended in inhibitor–doped medium, switching the inhibitor in half of the imatinib and ponatinib wells (Table 1). Finally, the cells were counted as before, and diluted to 10^5^ cells in 1mL medium once more in a fresh 24 well plate. This procedure was repeated 6 times, for a total of 36 days and three cycles in the drug rotation wells. As a control, cells were grown without inhibitors and washed every 3 days with the same procedure.

Note that in a quarter of the replicates cell counts were performed manually on a haemocytometer, using a 1–4:1 dilution with trypan blue depending on the density, and were diluted after washing assuming that 20% of the cells were lost, rather than being recounted.

**Table 1:** Drug rotation exposure schedule.

Days	Drug rotation, imatinib first	Drug rotation, ponatinib first	Imatinib monodrug	Ponatinib monodrug
1–6	Imatinib	Ponatinib	Imatinib	Ponatinib
7–12	Ponatinib	Imatinib	Imatinib	Ponatinib
13–18	Imatinib	Ponatinib	Imatinib	Ponatinib
19–24	Ponatinib	Imatinib	Imatinib	Ponatinib
25–30	Imatinib	Ponatinib	Imatinib	Ponatinib
31–36	Ponatinib	Imatinib	Imatinib	Ponatinib

### Data analysis

Dose–response relationships were calculated using R scripts and the drc package. A time dependent growth rate for all cells over time was calculated using ratrack^25^, both separately for each individual culture, and grouped by treatment. The carrying capacity of KCL-22 cells was estimated to be 3 × 10^6^ cells/mL.

### Simulations

Simulations of cell population developing resistance were run using the wollsey package^22^. The protocol was the same as in^22^. Briefly, given a starting population of cells that do not carry any resistance mutations and a table listing mutations and their IC50 values, the development of mutations under treatment is followed stochastically. A treatment protocol is also included. Given that we model an active form of cancer, the number of cancer stem cells in the simulation was kept roughly constant at 1 × 10^6^ cells. The probability for mutation was set to 1 × 10^−7^ per base pair, consistent with estimations in blood cancers and about two order of magnitude larger than the mutation rate in normal cells.

## Supplementary Information

Supplementary Information.
