# Supplementary material for: Rotating between ponatinib and imatinib temporarily increases the efficacy of imatinib as shown in a chronic myeloid leukaemia model
Source: Sci Rep. 2022 Mar 25;12:5164. doi: 10.1038/s41598-022-09048-5 (PMC8956613; doi:10.1038/s41598-022-09048-5)
Supplement: Supplementary file 1 — Supplementary Information. [file 41598_2022_9048_MOESM1_ESM.pdf]

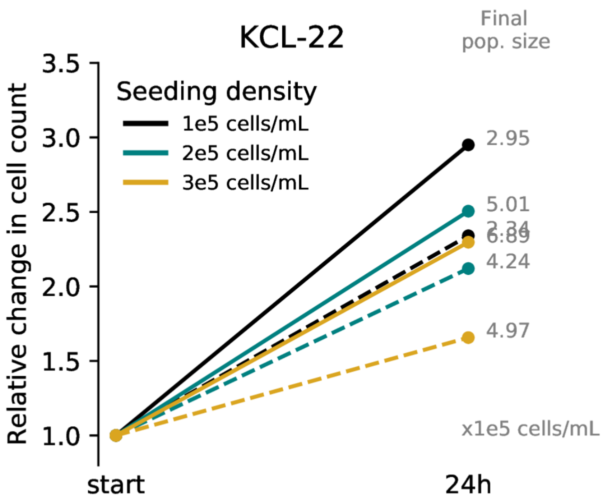

Figure S1: KCL-22 cells were seeded at three different densities in 24-well plates. After 24 h they were counted using a LUNA-II cell counter. The difference in relative growth was not significant between the seeding densities (χ^2-test, KCL-22: p=0.61). For growth curves in the absence of drugs see Figure S4.

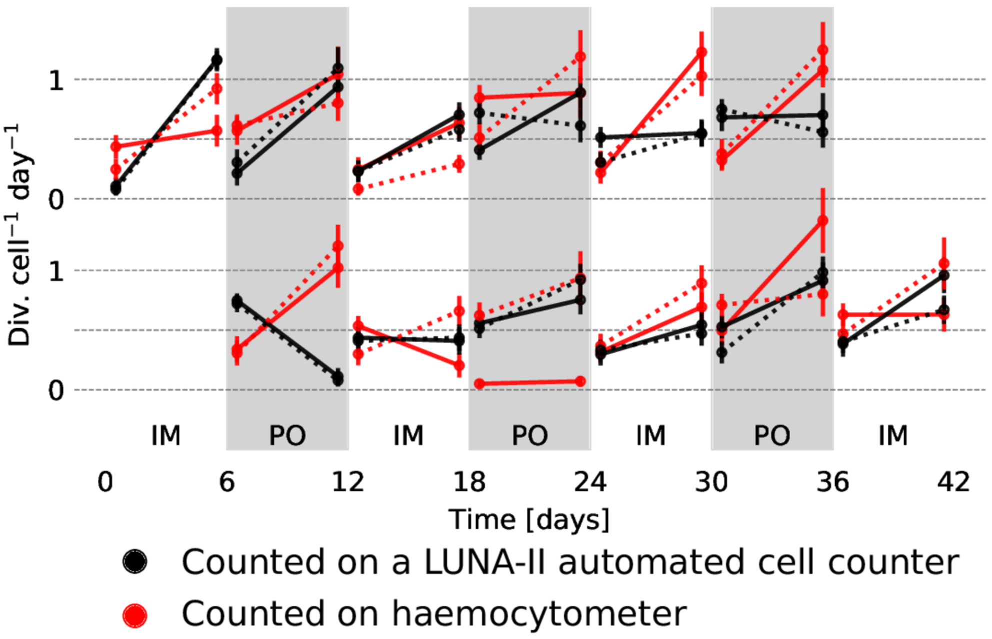

Figure S2: The inferred growth rate over time for KCL-22 cells without any grouping. The error bars show standard deviations.

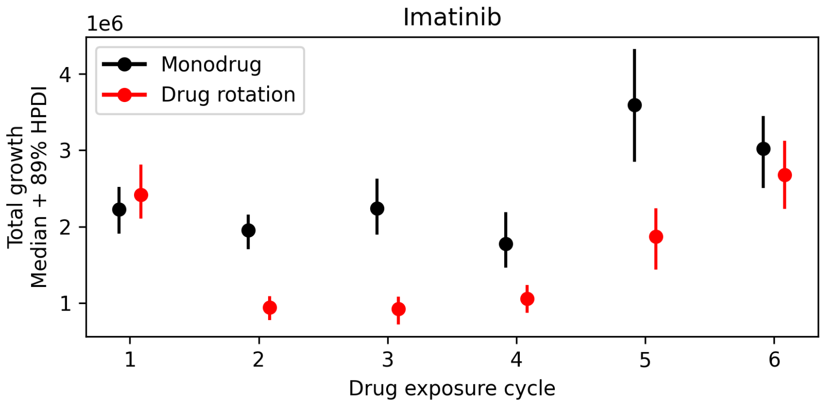

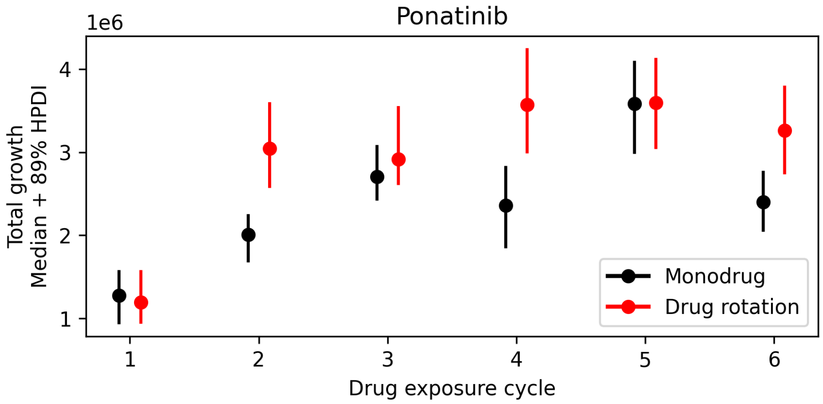

Figure S3: Total growth median + 89% highest posterior density interval (HPDI) as calculated for KCL-22 cells treated with imatinib first (top) or ponatinib first (bottom). The HPDI can be used in manner similar to the confidence interval (CI). The difference between these is that HPDI is derived from a posterior sampling approach. Non-overlapping credible intervals are an indicator that it is unlikely the two intervals originate from the same distribution. Calculated from the data shown in Figure 3. The data enables accurate calculation of the 89% HPDI but not 95% HPDI. Note that the drug rotation datapoints in each plot originate from two separate growth timelines: the drug rotation starting with imatinib, and the drug rotation starting with ponatinib, and have been interleaved based on the exposure week. For example, in the imatinib plot, the red interval at cycle 2 comes from day 12-18 in the bottom row of Figure 3A.

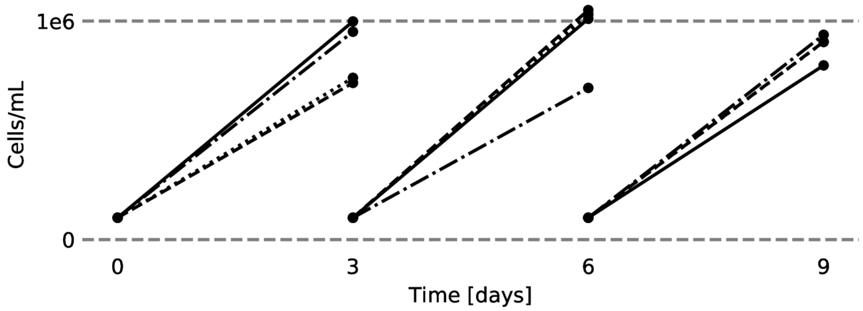

Figure S4: The number of cells (population size) over time for cultures grown without inhibitors. The reseeding involved an identical washing procedure to the treated cells.
